# Successful removal of a migrated plastic stent using a new endoscopic sheath

**DOI:** 10.1055/a-2215-1232

**Published:** 2023-12-13

**Authors:** Manabu Yamada, Takeshi Okamoto, Naoki Sasahira

**Affiliations:** 1 Department of Hepato-Biliary-Pancreatic Medicine, Cancer Institute Hospital of Japanese Foundation for Cancer Research, Koto-ku, Japan

Migration of plastic stents after endoscopic biliary stenting (EBS) occurs in 5%–10% of patients 1 . We report successful removal of a migrated plastic stent using EndoSheather (Piolax, Inc., Kanagawa, Japan), a new endoscopic sheath designed to facilitate targeted biopsies of the biliary tree 2 .

A 58-year-old woman with a history of EBS due to malignant biliary obstruction secondary to pancreatic cancer presented with fever and jaundice. Computed tomography revealed proximal migration of the plastic stent ( Fig. 1 ). Endoscopic retrograde cholangiopancreatography (ERCP) was performed for stent removal and biliary drainage ( Video 1 ).

**Fig. 1:**
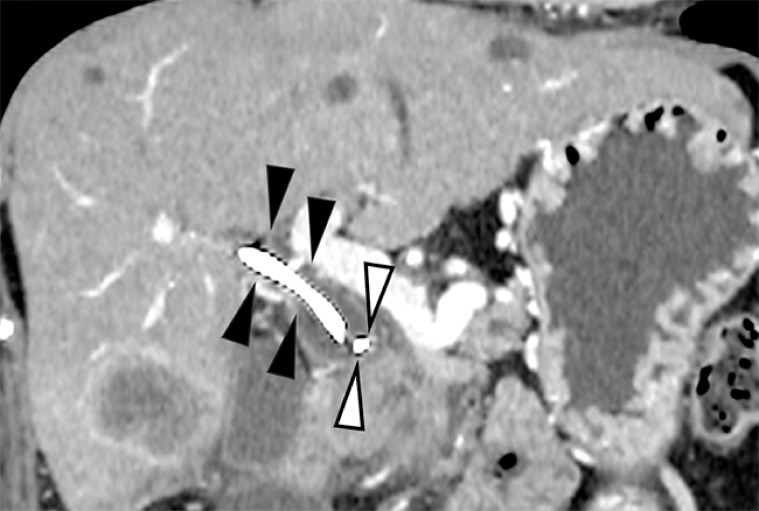
Contrast-enhanced computed tomography revealed migration of a plastic stent (black arrowheads), with the distal end located above the stricture in the distal common bile duct. The proximal flap of the plastic stent is also seen (white arrowheads).

Successful removal of a migrated plastic stent using a new endoscopic sheath.Video 1

Fluoroscopy confirmed proximal migration of an 8.5-Fr, 5-cm straight plastic stent. Stent removal with biopsy forceps was first attempted but proved unsuccessful. An EndoSheather was then inserted into the common bile duct along a 0.025-inch guidewire. After removal of the inner catheter, forceps were advanced through the outer sheath. After slight pushing of the scope to align the axes of the migrated stent and the forceps, the proximal flap of the stent was grasped with the forceps ( Fig. 2 ). The stent was successfully removed together with the EndoSheather and a double pigtail plastic stent was placed across the papilla. No adverse events occurred.

**Fig. 2:**
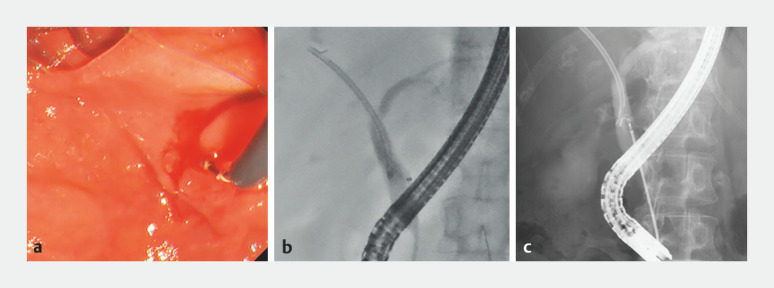
EndoSheather is a new endoscopic sheath with an outer sheath diameter of 2.4mm. The lumen of the outer sheath (after the inner sheath is removed) measures 2.1mm in diameter, allowing various devices up to 1.9mm (5.7Fr) in diameter to be inserted with ease. The inner sheath, made of high-density polyethylene, has a tapered tip that facilitates passage through stenoses. The EndoSheather has only been approved for use in Japan. **b** The tip of the EndoSheather was advanced to near the distal end of the plastic stent. **c** The proximal flap was successfully grasped by endoscopic forceps.

Options for removal of migrated biliary stents include basket catheters, balloon catheters, and forceps, but success rates are suboptimal. The EndoSheather has a tapered catheter tip that facilitates passage through stenoses and allows safe advancement of forceps to the desired location within the biliary tree. It also allows stent removal without requiring a guidewire to be maneuvered through the stent, as is necessary for stent removal using a drill dilator 3 . There have also been reports of the EndoSheather being used for removal of a migrated plastic stent 4 and for stent deployment during endoscopic ultrasound-guided biliary drainage 5 .

Endoscopy_UCTN_Code_TTT_1AR_2AZ

## Notes

Endoscopy E-Videos https://eref.thieme.de/e-videos
*E-Videos* is an open access online section of the journal *Endoscopy* , reporting on interesting cases and new techniques in gastroenterological endoscopy. All papers include a high-quality video and are published with a Creative Commons CC-BY license. Endoscopy E-Videos qualify for HINARI discounts and waivers and eligibility is automatically checked during the submission process. We grant 100% waivers to articles whose corresponding authors are based in Group A countries and 50% waivers to those who are based in Group B countries as classified by Research4Life (see: https://www.research4life.org/access/eligibility/ ). This section has its own submission website at https://mc.manuscriptcentral.com/e-videos .
